# Effects of hunting on cougar spatial organization

**DOI:** 10.1002/ece3.1089

**Published:** 2014-05-05

**Authors:** Benjamin T Maletzke, Robert Wielgus, Gary M Koehler, Mark Swanson, Hilary Cooley, J Richard Alldredge

**Affiliations:** 1 Large Carnivore Conservation Lab, School of the Environment, Washington State UniversityPullman, Washington, 99164; 2 Washington Department of Fish and Wildlife600 Capitol Way North, Olympia, Washington, 98501; 3 Department of Statistics, Washington State UniversityPullman, Washington, 99164

**Keywords:** Cougar, home range, hunting, *Puma concolor*, spatial organization, territoriality

## Abstract

The effects of increased mortality on the spatial dynamics of solitary carnivores are not well understood. We examined the spatial ecology of two cougar populations that differed in hunting intensity to test whether increased mortality affected home range size and overlap. The stability hypothesis predicts that home range size and overlap will be similar for both sexes among the two areas. The instability hypothesis predicts that home range size and overlap will be greater in the heavily hunted population, although may differ for males versus females due to behavior strategies. We marked 22 adult resident cougars in the lightly hunted population and 20 in the heavily hunted population with GPS collars from 2002 to 2008. Cougar densities and predation rates were similar among areas, suggesting no difference in per capita resources. We compared home range size, two-dimensional home range overlap, and three-dimensional utilization distribution overlap index (UDOI) among annual home ranges for male and female cougars. Male cougars in the heavily hunted area had larger sized home ranges and greater two-dimensional and three-dimensional UDOI overlap than those in the lightly hunted area. Females showed no difference in size and overlap of home range areas between study populations – further suggesting that differences in prey quantity and distribution between study areas did not explain differences in male spatial organization. We reject the spatial stability hypothesis and provide evidence to support the spatial instability hypothesis. Increased hunting and ensuing increased male home range size and overlap may result in negative demographic effects for cougars and potential unintended consequences for managers.

## Introduction

For the last 50 years, management of cougars in western North America has focused on setting harvest for recreational value and to protect livestock, human safety, sensitive species, or bolster ungulate populations (Logan and Sweanor 2010; Jenks 2011) because it is generally believed that increased harvest will reduce the density of cougars. Management strategies that incorporate metapopulation (source–sink) dynamics can be used to accomplish harvest objectives while ensuring overall population viability (Laundré and Clark 2003). Source areas are generally located in areas with limited access such as designated wilderness areas, national parks, monuments, and sinks in areas where access is high, cougar predation is a concern, or cougar-human conflicts occur (Laundré and Clark 2003). However, source–sink management may not account for mortality effects on demography and spatial dynamics or the unintended consequences on management objectives when harvest is spatially clumped (Beausoleil et al. 2013).

**Figure 1:**
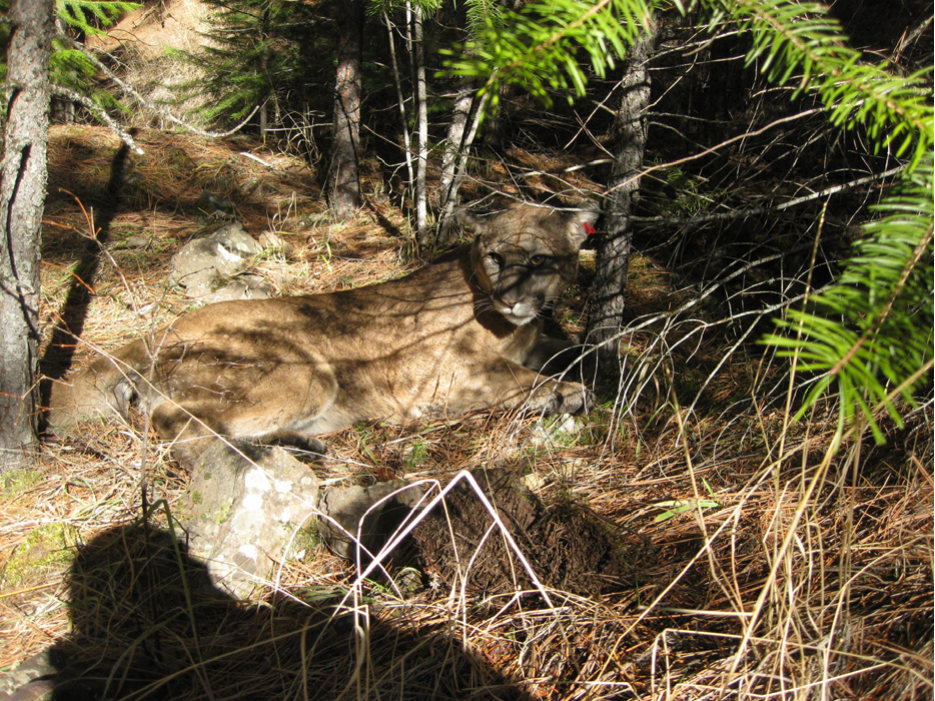
Photograph of an adult male cougar recovering after being immobilized and collared as part of a long-term research project.

Long-term research has provided insights into the negative effects of high (>20% per year) mortality on demographics of cougar populations (Fig. 1; Laundré et al. 2000; Robinson et al. 2008; Cooley et al. 2009a,b; Ruth et al. 2011; Newby et al. 2013; Wielgus et al. 2013). However, little is known about the effects of high mortality on cougar spatial dynamics and social organization.

Cougar populations are thought to be regulated by competition for food in females, and competition for mates in males (Logan and Sweanor 2001, 2010). Because of differing regulating mechanisms for each sex, we would expect differences in response to increased mortality. Female home range size and intrasexual overlap is expected to remain unchanged with increasing female mortality because they are food-limited (Pierce et al. 2000; Logan and Sweanor 2001). We expect when an adult male is removed in low-harvest scenarios, the home range boundaries remain until a new adult male fills the void because of territorial behavior of adjacent males (Hornocker 1969; Seidensticker et al. 1973; Logan and Sweanor 2001). If mortality is sufficient to reduce several adjacent males, then territorial boundaries cease to exist in that unit of space until other males fill the void. We expect the new boundaries established by immigrant males that are unfamiliar with the area to be more fluid and larger in search of breeding opportunities and prey without initial competition of other males. Therefore, high male mortality should result in spatial instability (larger home ranges and overlaps) in male cougars. In a high-harvest scenario where several adjacent adult males are killed annually, territorial boundaries break down, resulting in spatial instability (larger home ranges and overlap) until new boundaries are established by immigrant males.

We tested the spatial stability (no hunting effect) versus the spatial instability (hunting effect) hypotheses for cougars by comparing spatial ecology among two cougar populations subjected to low and high-harvest intensities. Both populations had similar densities and per capita predation rates (Cooley et al. 2009b). The stability hypothesis predicts that home range size and overlap will be similar for both sexes among the two areas. The instability hypothesis predicts that home range size and overlap will be greater in the heavily hunted population, although may differ for males versus females due to behavior strategies to maximize individual reproductive success (Logan and Sweanor 2001).

## Study Areas

### Heavily hunted area

The heavily hunted study area located near Kettle Falls, WA (48°N, 118°W), was 1476 km^2^ and included a patchwork of federal, state, and privately owned lands. The elevation varied from 400 to 2130 m and occupied the transition between the East-slope Cascades and Northern Rocky Mountain physiographic province (Bailey et al. 1994). Tree species included Douglas-fir (*Pseudotsuga menziesii*), western hemlock (*Tsuga heterophylla*), ponderosa pine (*Pinus ponderosa*), western red cedar (*Thuja plicata*), and subalpine fir (*Abies lasiocarpa*). Most of the 46 cm annual precipitation fell as snow from mid-November through April. Mean annual temperatures ranged from −6°C in January to 21°C in July. White-tailed deer (*Odocoileus virginianus*) were the most abundant ungulate, but mule deer (*Odocoileus hemionus*), elk (*Cervus elaphus*), and moose (*Alces alces*) are also present. Predator species included coyote (*Canis latrans*), black bear (*Ursus americanus*), and bobcat (*Lynx rufus*). Cougar hunting without the use of hounds was permitted in the study area each year from 1 September to 30 November. Hunting with the aid of hounds occurred from 1 December to 31 March.

### Lightly hunted area

The lightly hunted study area near the town of Cle Elum, WA (47°N, 121°W), was 1652 km^2^ and was located along the east slope foothills of the North Cascades foothills. The majority of the study area was a patchwork of U.S. Forest Service, privately owned timber lands, residential, and agricultural areas. Elevation ranged from 462 to 2279 m. Sagebrush steppe foothills (below 550 m elevation) transition to ponderosa pine and Douglas-fir covered slopes. Subalpine fir, Engelmann spruce (*Picea engelmannii*), Pacific silver fir (*Abies amabilis*), and western hemlock dominate elevations from 1550 to 2279 m. Precipitation averaged 56.4 cm/years. Mean annual temperature ranged from −7°C in January to 27°C in July. Elk and mule deer occurred throughout the study area, and mountain goats (*Oreamnos americanus*) were present at higher elevations. Other predator species included coyote, black bear, and bobcat. Cougar harvest without the use of hounds was permitted in the study area each year from 1 August to 15 March.

### Demographic comparisons of the study areas

Cooley et al. (2009b) estimated an overall annual hunting mortality rate of 0.24 (±0.05 SD), an adult male hunting mortality rate of 0.35 (±0.08 SD), and an adult female hunting mortality of 0.16 (±0.05 SD) in the heavily hunted area. The survival-fecundity rate of growth was 0.78 (±0.19 SD), and net immigration rate was 0.13, resulting in an observed growth rate of 0.91 (Cooley et al. 2009b). Mean age of independent radio-collared cougars was 27 months (±4 SD). Total density remained relatively stable via immigration for 5 years at 3.46 (±0.69 SD) cougars/100 km^2^ (Cooley et al. 2009b). The predation rate for all independent radio-collared animals was 6.68 days between ungulate kills (Cooley et al. 2009b).

Cooley et al. (2009a,b2009b) estimated an overall mortality rate from hunting of 0.11 (±0.04 SD), an adult male mortality rate of 0.16 (±0.06 SD), and an adult female hunting mortality of 0.07 (±0.05 SD) in the lightly hunted area. The survival-fecundity rate of growth was 1.10 (±0.12 SD), and net emigration rate was 0.12 resulting in an observed growth rate of 0.98 (Cooley et al. 2009b). Mean age of independent radio-collared cougars was 39 months (±4 SD). Total density of all cougars remained relatively stable via emigration for 5 years at 3.62 (±0.58 SD) cougars/100 km^2^ (Cooley et al. 2009b). The predation rate for all independent radio-collared animals was 7.04 days between ungulate kills (Cooley et al. 2009b).

## Methods

### Captures and monitoring

We attempted to capture and mark all cougars each year, from 2001 through 2008, by conducting thorough and systematic searches for tracks in winter in each study area (Robinson et al. 2008 and Cooley et al. 2009a,b2009b). We used trained dogs to track and tree cougars (Hornocker 1969), and immobilized cougars with ketamine hydrochloride (200 mg/mL) and xylazine hydrochloride (20 mg/mL) at a dosage of 0.4 mL/10 kg of body mass, or with Telazol at a dosage of 6 mg/kg, using a projectile dart (Dan-inject, Børkop, Denmark; Ross and Jalkotzy 1992, Spreadbury et al.^1996^). We determined sex and classified animals as kittens (0–12 months), juveniles (13–24 months), or adults (>24 months) based on physical measurements and canine tooth gum regression (Laundré et al. 2000); however, we only used resident adults >24 months for the analyses. We fitted each adult animal with a Global Positioning System radio collar (GPS; Lotek Wireless, Newmarket, Ontario, Canada and Televilt, Lindesberg, Sweden). Collars were programmed to collect locations at 4-h intervals, and data were retrieved using a remote communication unit. Collar acquisition rate was 64% with collar positional errors <25 m (Di Orio et al. 2003). We handled all animals in accordance with Washington State University Animal Care (IACUC Permit #3133) and Animal Welfare Assurance Committee (AWAC Permit #A3485-01).

### Home range size

We calculated fixed kernel home range (99% volume) for each adult radio-collared cougar each year (Seaman et al. 1999; Kertson et al. 2013) because the accuracy of the GPS data (<25 m) allowed us to use all locations for each animal's home range estimation. We did not analyze reproductive status (females with/without offspring) separately because of sample size constraints.

We used the Adehabitat package (Calenge 2006) in program R (R Development Core Team 2010) to calculate the plug-in smoothing parameter (“hpi”) for the 99% kernel density estimate (Gitzen and Millspaugh 2003; Gitzen et al. 2006). We entered the hpi value calculated for each cougar into Hawth's tools extension in ArcGIS 9.3 to calculate a kernel density function (30 m cell resolution) and the 99% volume contour (Beyer 2004). We calculated each animal's utilization distribution by dividing each cell value in the density grid by the sum of all cell values in each kernel density grid. We then created a 99% volume contour polygon from the utilization distribution for each cougar and calculated the home range area in square kilometers.

### Home range overlap

Home range overlap is an effective measure of shared space use for territorial species and is useful for assessing the degree of interaction among individuals (Kernohan et al. 2001; Marzluff et al. 2001; Millspaugh et al. 2004; Fieberg and Kochanny 2005). This method has been used to assess spatial organization in a number of carnivore species including cougars (Seidensticker et al. 1973; Logan and Sweanor 2001), jaguars (*Panthera onca*; Rabinowitz and Nottingham 1986), bobcats (*lynx rufus*; Nielsen and Woolf 2001), and coyotes (*Canis latrans*; Attwood and Weeks 2003). Two-dimensional overlap quantifies the direct home range overlap of two animals and determines how much space they share relative to their home range size. The three-dimensional overlap compares the heterogeneity of use within each animal's home range where they overlap and quantify how much they use the shared space.

We calculated home range overlap for each year for each independent adult (≥24 months) and summed the area shared with the “polygon in polygon” function in Hawth's tools in ArcGIS 9.3 (Fig. 1). The shared area was divided by the total home range size for each cougar. We averaged all home range overlaps between each individual cougar and all adjacent cougars.

Two-dimensional polygon space use does not consider the internal heterogeneity of use within the home range (Kernohan et al. 2001). The utilization distribution overlap index (UDOI) compares the three-dimensional utilization distribution for overlapping home ranges (Fieberg and Kochanny 2005). We used the UDOI for quantifying overlap of shared space use (Fieberg and Kochanny 2005) and the “kerneloverlap” function in Adehabitat (Calenge 2006) in program R to calculate the UDOI (Fieberg and Kochanny 2005).

### Statistical analysis

We used a factorial analysis of variance (Zar 1996) to test for differences between study areas, sexes, and interactions of study area by sex for home range size, two-dimensional home range overlap, and three-dimensional UDOI. We used a log-transformation to normalize the data for home range size, and arcsine square root transformation to approach normality for the two-dimensional overlap and UDOI data. To test for significant differences (*α* = 0.05) in home range size, two-dimensional overlap and UDOI between cougar demographic groups, we used the Fisher's least significant difference post hoc test with the Holm–Bonferroni adjustment to control the familywise error rate (Holm 1979). We used the average home range size and overlaps for individuals collared for multiple years so that each animal was an experimental unit.

## Results

### Captures

We captured, collared, and monitored 20 (seven males, 13 females adult cougars) in the heavily hunted area from December 2004–2007 for an average of 3.0 ± 0.8 males and 6.0 ± 2.2 females per year. From December 2001 to 2008, we captured, collared, and monitored 22 (13 males, nine females adult cougars) in the lightly hunted area for an average of 3.1 ± 0.6 males and 4.1 ± 1.4 females per year.

### Home range size

Home ranges and utilization distributions were calculated using an average of 760 ± 418 GPS acquisitions per year (*n* = 82). Variation in home range size was attributable to sex, study area, and a sex by study area interaction (Table 1). Male home range sizes were twice as large (753 km^2^ vs. 348 km^2^) (*P* < 0.01) in the heavily hunted versus the lightly hunted study area, but no difference (249 km^2^ vs. 199 km^2^) was observed in home range sizes for females (*P* = 0.53) (Table 2).

**Table 1:** Analysis of variance tests for log-transformed cougar home range areas by sex near Cle Elum, (lightly hunted) and Kettle Falls (heavily hunted), Washington, 2001–2008. Lilliefors K-S Normality test = 0.122, *P* = 0.115

Source	Type III SS	df	Mean Sq	*F*-ratio	*P*-value
Sex	6.789	1	6.789	42.31	<0.01
Study area	1.892	1	1.892	11.79	<0.01
Sex*Study area	1.053	1	1.053	6.57	0.01
Error	6.097	38	0.160		

**Table 2:** Annual fixed kernel home ranges (99% volume contour) for cougars near Cle Elum (lightly hunted) and Kettle Falls (heavily hunted), Washington, 2001–2008

	Lightly hunted			Heavily hunted				
				
Sex	*n*	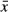 (km^2^)	SD	*n*	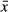 (km^2^)	SD	*P*-value*	*α*/*k*^1^
♂	13	347.5	134.4	7	752.5	337.5	<0.01	0.025
♀	9	198.9	42.9	13	240.2	103.7	0.53	0.05

* Fisher's least significant difference post hoc test.

1 Holm–Bonferroni adjusted alpha value where *α* = 0.05 and *k* is the number of pairwise comparisons.

**Figure 2:**
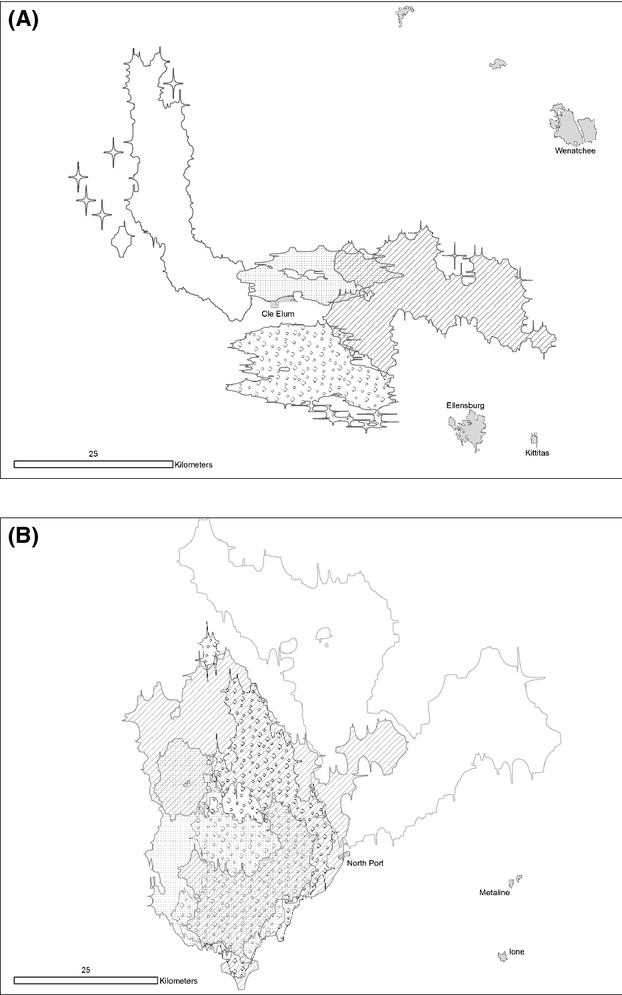
Examples of two-dimensional home range overlaps of four male cougar in Cle Elum, WA, (lightly hunted area) in 2008 (A) and the home ranges of four male cougar in near North Port, WA, in 2007 (B).

Variation in home range overlap was also attributable to sex, study area, and a sex by study area interactions. We observed a higher two-dimensional home range overlap (*P* < 0.01) for males in the heavily hunted population than in the lightly hunted population (Figure 2, Table 3). There were no differences in female to female, female to male, or male to female overlap between the two areas (Table 4).

**Table 3:** Analysis of variance tests of the area of overlap (arcsine square root transformed) for male and female cougars near Cle Elum, (lightly hunted) and Kettle Falls (heavily hunted), Washington, 2001–2008. Lilliefors K-S Normality test = 0.076, *P* = 0.04

Source	Type III SS	df	Mean Sq	*F*-ratio	*P*-value
Sex	2.863	3	0.954	18.23	<0.01
Study area	0.258	1	0.258	4.93	0.03
Sex*Study area	0.539	3	0.180	3.44	0.02
Error	7.015	134	0.052		

**Table 4:** Average two-dimensional overlap between adjacent cougars in Cle Elum (lightly hunted) and Kettle Falls (heavily hunted), Washington, 2001–2008

	Lightly hunted			Heavily hunted				
				
Sex	*n*	Overlap	SD	*n*	Overlap	SD	*P*-value*	*α*/*k*^1^
♂	19	0.17	0.11	9	0.41	0.23	<0.01	0.01
♀	24	0.20	0.15	19	0.31	0.18	0.03	0.02
♂ – ♀	20	0.26	0.18	9	0.16	0.06	0.22	0.03
♀ – ♂	29	0.51	0.26	13	0.57	0.19	0.55	0.05

* Fisher's least significant difference post hoc test.

1 Holm–Bonferroni adjusted alpha value to control for familywise error rates where *α* = 0.05 and *k* is the number of pairwise comparisons.

**Figure 3:**
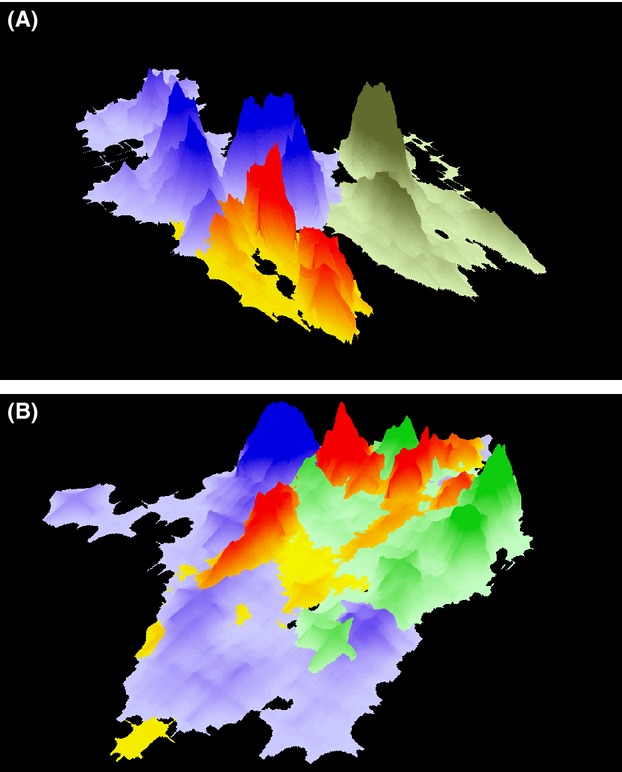
Home range utilization distributions for six male cougars: three (A) in the lightly hunted area near Cle Elum, 2008 and three (B) in the heavily hunted area near Kettle Falls, Washington, 2007. The color ramps represent individual cougars, and the peaks represent areas of high probability of use.

We found a significant interaction between sex and study area for three-dimensional UDOI overlap (Fig 3). The heavily hunted area had higher UDOI values for males (Table 5) than the lightly hunted area (0.38 vs. 0.16, *P* < 0.01) indicating more shared space use, but there were no differences in the female to female, female to male, and male to female UDOI (Table 6).

**Table 5:** Analysis of variance tests of utilization distribution overlap index (arcsine square root transformed) for male and female cougars near Cle Elum, (lightly hunted) and Kettle Falls (heavily hunted), Washington, 2001–2008. Lilliefors K-S Normality test = 0.042, *P* = 0.718

Source	Type III SS	Df	Mean Sq	*F*-ratio	*P*-value
Sex	0.429	3	0.14	2.259	0.08
Study area	0.104	1	0.10	1.650	0.20
Sex*Study area	0.687	3	0.23	3.622	0.02
Error	8.539	135	0.06		

**Table 6:** Three-dimensional home range overlap calculated using the utilization distribution overlap index (UDOI) for cougars in a Cle Elum (lightly hunted) and Kettle Falls (heavily hunted), Washington, 2001–2008

	Lightly hunted			Heavily hunted				
				
Sex	*n*	Overlap	SD	*n*	Overlap	SD	*P*-value*	*α*/*k*^1^
♂	19	0.16	0.15	9	0.38	0.27	0.01	0.01
♀	26	0.12	0.14	19	0.27	0.29	0.04	0.02
♂ – ♀	21	0.30	0.25	9	0.19	0.08	0.36	0.03
♀ – ♂	29	0.32	0.30	13	0.19	0.11	0.30	0.05

* Fisher's least significant difference post hoc test.

1 Holm–Bonferroni adjusted alpha value to control for familywise error rates where *α* = 0.05 and *k* is the number of pairwise comparisons.

## Discussion

Our data suggest a difference in male, but not female, cougar spatial organization as a result of high hunting mortality. Home range size, two-dimensional overlap, and three-dimensional UDOI overlap for males were 2–3 times greater in the heavily hunted area (Fig. 2). This contrasts with the lightly hunted area, where high-use areas (as indicated by UD values) were mutually exclusive between resident males. Total densities (3.46 vs. 3.62 cougars/100 km^2^), predation rates (6.68 vs. 7.04 days/kill), and home range size of females (240 ± 103 vs. 198 ± 42 km^2^) were similar between areas, suggesting that differences in prey availability did not explain differences in male home range size and overlap (Cooley et al. 2008, 2009b; White et al. 2011). Female home range sizes, two-dimensional, and three-dimensional overlaps were not significantly different between study areas (Table 4), and we observed an overlap of 12–31% with other females. We did not observe shifts in spatial distribution and overlap in female home ranges in response to mortalities where we did observe territory shifts for males.

Solitary male carnivores that breed year-round tend to increase their reproductive success by defending a territory from other males while overlapping with as many females as possible (Sandell^1989^). However, increased mortality of adult males causing frequent territorial voids and subsequent home range shifts may disrupt territoriality and stable spatial organization resulting in boundaries that may become fluid. Continued high mortality results in a continuous male turnover and prolonged spatial instability which may lead to the negative demographic effects observed by Lambert et al. (2006); Robinson et al. (2008); Cooley et al. (2009ab2009b); Ruth et al. (2011); and Wielgus et al. (2013).

By contrast, female cougar rear kittens by themselves and their reproductive success are related to their success in securing food (Logan and Sweanor 2001, 2010). Females may increase their reproductive success by occupying areas where prey is adequate and where they can establish familiarity with the landscape to enhance capture success (Pierce et al. 2000). Therefore, females may be better off maintaining a stable home range of sufficient size where prey is adequate for rearing young rather than expanding into new and unknown territory.

In addition to behavioral responses to different harvest rates, other factors that may influence home range size and overlap are collar types, use of different home range estimators, habitat and climatic variables, and prey availability. Topography, vegetation, and climate were similar, the GPS collars deployed were the same or of similar make and design, and the estimator used to calculate home range size for the two study areas was the same, negating these potential influences. In addition, prey base was similar between areas as prey availability and predation rates were similar (6.68 vs. 7.04 days/kill), Cooley et al. 2009a,b2009b; White et al. 2011).

### Implications for conservation and management

Management for the heavily hunted area was designed specifically to increase harvest to reduce cougar densities in order to reduce potential human conflicts (Washington Department of Fish and Wildlife 2008). However, with increased harvest, the unintended consequences may have resulted in increased cougar interactions with livestock, prey, and people (Peebles et al. 2013). As shown from this study, high harvest leads to larger sized and greater overlap for male home ranges, and densities may not decrease as intended. This results in greater numbers of subadult males overlapping a unit of space and potentially increasing encounter rates with people, pets, or livestock. As Kertson et al. (2013) found, subadult cougars utilized areas near residential development more than adults, thereby increasing potentials for conflicts and sightings. Such records of human interactions or sightings used to index densities and population trends may mislead managers into an inappropriate harvest response.

Wielgus et al. (2013) observed that male mortality may actually be dispensatory resulting in increased infanticide and reduced population growth for females. Evidence of infanticide is rare in areas with low mortality where cougar territory boundaries are stable and a resident male overlaps several females' home ranges over a period of years (Ross and Jalkotzy 1992, Logan and Sweanor 2001; Cooley et al. 2009a,b2009b). In contrast where mortality of adult males is high, there is an influx of immigrant subadult males with larger home range sizes and greater overlap for males. These situations may result in higher rates of mortality of females as they defend their young and infanticide may increase, resulting in reduced female and kitten survival (Logan and Sweanor 2001; Cooley et al. 2009b; Ruth et al. 2011).

The large home range areas for males in a heavily harvested area may be interpreted as a metric of poor habitat quality (Julian et al. 2012), when in fact it may not indicate a resource-poor habitat where prey and/or numbers of females are low but may be an indicator of an overexploited population.

Managers should strive to conserve a proportion of older individual males (Whitman et al. 2004) in populations to maintain spatial stability, which may help to minimize the unintended consequences of high harvest (Packer et al. 2009). Washington State instituted regulations in 2013 to reduce the harvest threshold below the intrinsic growth rate of 14% (Beausoleil et al. 2013) in order to maintain home range and spatial stability for cougar populations.
